# Rotational Atherectomy in Coronary Heart Disease Patients with Different Rotational Speed: In Hospital and Six-Month Outcomes

**DOI:** 10.31083/j.rcm2401013

**Published:** 2023-01-06

**Authors:** Jiawei Wu, Guangquan Qiu, Hao Hu, Li-Kun Ma

**Affiliations:** ^1^Department of Cardiology, The First Affiliated Hospital of USTC, Division of Life Science and Medicine, University of Science and Technology of China, 230001 Hefei, Anhui, China; ^2^Department of Cardiology, Anhui Provincial Hospital Affiliated to Anhui Medical University, 230001 Hefei, Anhui, China

**Keywords:** coronary heart disease, rotational atherectomy, rotational speed

## Abstract

**Background:**

Rotational atherectomy (RA) is an important technique for the management of severe coronary calcification. However, optimal rotational speed is yet to be defined.

**Methods:**

A total of 372 coronary heart disease (CHD) patients were retrospectively analyzed between February 2017 and January 2022. The patients were divided into four groups based on the maximum RA speed: group 1 (<150,000 rpm, 76 cases), group 2 (150,000 rpm, 156 cases), group 3 (160,000 rpm, 90 cases) and group 4 (≥170,000 rpm, 50 cases). The outcomes analyzed were procedural complications, six-months major cardiovascular and cerebrovascular events (MACCE) and chronic heart failure.

**Results:**

Patients in group 4 had a higher incidence of slow flow during the RA operation (*p* = 0.025). There was no significant difference in other complications among the four groups, as well as six-month MACCE. After adjusting for confounding factors, increase in rotational speed led to a higher probability of slow flow (*p *for non-linearity = 0.131; adjusted model) and MACCE (*p* for non-linearity = 0.183; adjusted model). Logistic regression analysis showed that rotational speed was a predictor of slow flow during RA operation (OR = 1.25, 95% CI: 1.05~1.49, *p* = 0.01). Moreover, the analysis demonstrated that individuals with lower rotational speed (<150,000 rpm) were at 230% higher risk of vasospasm compared with a higher rotational speed (160,000 rpm) (OR = 3.3, 95% CI: 1.08~10.09, *p* = 0.036).

**Conclusions:**

CHD patients treated with a rotational speed of ≥170,000 rpm had a higher risk of slow flow after RA. Rotational speed is an independent risk factor for slow flow in CHD patients. Moreover, a rotational speed of <150,000 rpm was associated with a higher risk of vasospasm compared with rotational speed of 160,000 rpm. There was no significant difference in six-month outcomes in comparison to elective CHD patients with different rotational speeds, and the probability of MACCE was intensified with increase in rotational speed.

## 1. Introduction

With the expansion of the social economy and the aging population, the incidence of coronary heart disease (CHD) is increasing year by year, and the proportion of coronary calcification lesions has dramatically increased. Since early 1990s, rotational atherectomy (RA) has been the mainstay tool used to effectively debulk and modify calcified plaques, increasing the lumen area, facilitating the delivery of intraoperative balloons and stents, as well as improving stent expansion and apposition, thereby increasing the success rate of percutaneous coronary intervention (PCI) [1, 2, 3]. The rotation speed varies considerably among different interventional cardiologists (140,000 to 220,000 rpm) in clinical practice [4, 5, 6]. Moreover, there is limited data on the relationship between RA speed and long-term prognosis in patients with CHD, and determination of the optimal rotation speed remain controversial.

This study investigated interventional outcomes of RA at different rotational speeds and analyzed clinical outcomes in patients with CHD.

## 2. Methods

### 2.1 Study Population

This observational, retrospective study was conducted between February 2017 and December 2021. The study enrolled a total of 372 CHD patients with severe coronary calcification who were treated with RA. The patients were divided into four groups according to the maximum RA speed: group 1 (<150,000 rpm), group 2 (150,000 rpm), group 3 (160,000 rpm) and group 4 (≥170,000 rpm). Patients with severe helical dissection on angiography and those where the rotawire failed to pass were excluded from the study.

Demographic and clinical characteristics of the patients, including age, sex, and comorbidities such as a history of cardiovascular diseases (CVD) or stroke were collected. Some auxiliary examination data such as serum creatinine or fasting plasma glucose (FPG) were also collected. Lesion characteristics and procedural details, as well as any complications during RA operation and six-month outcomes were analyzed.

This study protocol was approved by the Medical Research Ethics Committee of the First Affiliated Hospital of USTC (Anhui Provincial Hospital) (No. 2022-RE-063).

### 2.2 Rotational Atherectomy

Our center used the Siemens DTC (or PHILIPS FD1010) angiography system for coronary angiography and interventional therapy. The Rotablator System (Boston Scientific Corporation, Natick, MA, USA) was used to perform all the RA procedures. Prior to the procedure, the patients received aspirin (100 mg/daily) and a thienopyridine (clopidogrel 75 mg/daily or ticagrelor 100 mg bid). In addition, intravenous unfractionated heparin (70 to 100 units/kg) was used to achieve an appropriate activated coagulation time (250 seconds) during the PCI. PCI was performed at the operator’s discretion based on the characteristics of the patient’s specific lesions and peripheral vascular access conditions. Guiding catheters up to 7F in diameter were used. With the help of a Finecross or Crossair microcatheter, a 0.009 in (1 in = 2.54 cm) rotawire floppy was exchanged to the distal of the target lesion. Each RA session lasted less than 30 seconds, and the interval between each RA was 60 seconds. During the RA procedure, unfractionated heparin (UFH) nitroglycerine flushing solution was continuously instilled under pressure. Non-compliant balloon dilation and stent placement were performed after RA based on the characteristics of the lesions. Because our experienced surgeons were less likely to experience bradyarrhythmias when using small burrs and lower rotational speeds, temporary pacemakers are rarely used. Patients with bradyarrhythmia after RA were either instructed to cough forcefully or administered with intravenous atropine. Atropine and temporary pacemakers were prepared in advance for patients with a dominant right coronary artery or circumflex. The decision to insert an intra aortic balloon Pump (IABP) was left to the discretion and guidance of the supervising cardiologists.

### 2.3 Endpoints

Perioperative endpoints in our study included occurrence of hypotension, vasospasm, dissection, slow flow, perforation, bradyarrhythmia, burr entrapment, rotawire fracture during RA, as well as the incidence of heart failure, stent thrombosis, and cardiac death during hospitalization. Long-term primary endpoint was six-month occurrence of major cardiovascular and cerebrovascular events (MACCE), which include a composite of myocardial infarction (MI), stent thrombosis, target vessel revascularization (TVR), cardiogenic death, all-cause death, and stroke. On the other hand, long-term secondary endpoint was six-month chronic heart failure.

### 2.4 Definitions

Severely calcified lesions can be assessed visually by coronary angiography (defined as radiometric turbidity without cardiac movement before contrast medium injection) or (intravascular ultrasound) IVUS showing superficial calcification involving more than 3 quadrants. Planned RA was defined as RA performed directly before balloon predilation, while bail RA was performed after balloon predilation failure or stent delivery to the target lesion. Procedural success was defined as final stenosis of less than 30%, with a Thrombolysis in Myocardial Infarction (TIMI) flow grade of 3. The procedure was considered a failure if patients received emergent coronary artery bypass grafting (CABG) and/or PCI, or other severe RA-related complications (coronary perforation, death) developed before discharge. Hypotension was defined by transient drop in blood pressure to 90/60 mmHg or a 20% drop from the original blood pressure level. Vasospasm was characterized by transient total or sub-total occlusion of epicardial arteries or severe diffuse vasoconstriction. Dissection referred to separation of true and false lumens that appear on angiography. Slow flow/no re-flow was defined as less than TIMI III flow grade in the absence of dissection or thrombus immediately after RA [7]. Coronary perforation referred to marked extravasation of contrast media or blood from the coronary artery visible on angiography during or following the interventional procedure [8]. Bradyarrhythmia was defined as transient sinus arrest, overt sinus bradycardia (heart rate <40 beats/min), and second-degree or greater atrioventricular block. Burr entrapment was defined by rapid drop in rotational speed with stuck and unmovable burr. Rotawire fracture was defined as structural separation of the head segment of the rotawire visible on angiography. In-hospital heart failure was defined as deterioration in signs and symptoms of in patients with previous chronic heart failure or new-onset heart failure requiring urgent therapy. Diagnostic criteria was based on an intravenous administration of diuretic drugs, vasodilators, or inotropic drugs, and including at least one of the followings: cardiac pulmonary edema or pulmonary vascular congestion on chest radiograph; heart failure causes one third of the lungs to rales; left ventricular end-diastolic pressure >18 mmHg; or dyspnea, with a Po2 <80 mmHg or an oxygen saturation <90% without oxygen inhaled (significant lung disease excepted); or the N-terminal pro brain natriuretic peptide increased beyond the upper limit of the reference value. Stent thrombosis was defined according to the Academic Research Consortium criteria [9]. Cardiogenic death was defined as unclear death but due to a noncardiac reason.

### 2.5 Statistical Analysis

Means (standard deviation) and number (proportions) were calculated according to RA speed categories. To compare characteristics of the study subjects between the RA speed categories, we employed analysis of variance for continuous variables and chi-squared or Kruskal-Wallis rank-sum test for categorical variables. Multiple unconditional logistic regression analyses were performed to estimate the relationship between the RA speed and primary endpoints with adjustments for potential confounders. In addition, odds ratios (OR) and 95% confidence intervals (95% CI) as well as regression models for different clinical subgroups were conducted. All *p* values were 2-tailed, with a significance threshold of 0.05. Data management and analyses were performed using R software (4.0.2, Vienna, Austria).

## 3. Results

A total of 372 CHD patients with severe coronary calcification were treated with RA, and included 76 (20%) patients in group 1 (<150,000 rpm), 156 (42%) in group 2 (150,000 rpm), 90 (24%) in group 3 (160,000 rpm) and 50 (13%) in group 4 (≥170,000 rpm). We analyzed baseline demographics, comorbidities, laboratory results, lesion, and procedural characteristics of the patients in the four groups as shown in Table 1. The proportion of patients with stable coronary heart disease (SCAD) and acute coronary syndrome (ACS) was 23.9% and 76.1%, respectively. The analysis showed that 80.3% of patients in group 1 had unstable angina (UA) compared to 65.4%, 55.6% or 56% UA cases in group 2, group 3 or group 4 (*p* = 0.005). Group 3 had a significantly higher number (20%) of non-ST-segment elevation myocardial infarction (NSTEMI) patients compared to 6%, 10.3% and 10% in group 1, group 2 and group 4, respectively (*p* = 0.005). The prevalence of other traditional cardiovascular risk factors, as well as comorbidities and auxiliary examination, were comparable in the four groups. As shown in Table 2, there were no significant differences in the procedural characteristics, including the target vessel for rotational atherectomy, the use of intra-aortic balloon pumps, the number of burrs used, initial burr-to-artery ratio, or the maximum burr-to-artery ratio among the four groups. More patients in group 4 experienced higher speed than initial speed. Of note, there were significant differences in TIMI flow grade following RA between the four groups, with patients in group 4 experienced higher incidence of slow flow. A total of 368 (98.9%) patients underwent drug-eluting stenting after RA, while no significant differences in the number of stents used were observed.

**Table 1.** **Patient characteristics among the different groups**.

Variables	Total (n = 372)	Group 1 (n = 76)	Group 2 (n = 156)	Group 3 (n = 90)	Group 4 (n = 50)	*p* value
Clinical presentation						
SCAD, n (%)	89 (23.9)	13 (17.1)	37 (23.7)	22 (24.4)	17 (34)	0.191
UA, n (%)	241 (64.8)	61 (80.3)	102 (65.4)	50 (55.6)	28 (56)	0.005
NSTEMI, n (%)	41 (11.0)	2 (2.6)	16 (10.3)	18 (20)	5 (10)	0.005
STEMI, n (%)	1 (0.3)	0 (0)	1 (0.6)	0 (0)	0 (0)	1.000
Patients characteristics						
Male, n (%)	217 (58.3)	48 (63.2)	97 (62.2)	44 (48.9)	28 (56)	0.165
Age, Median (IQR)	72.0 (66.0, 78.0)	73.0 (67.0, 78.0)	72.0 (65.8, 78.0)	71.5 (67.2, 78.0)	70.0 (66.0, 76.0)	0.559
CVD history, n (%)	334 (89.8)	68 (89.5)	145 (92.9)	76 (84.4)	45 (90)	0.211
Stroke history, n (%)	98 (26.3)	21 (27.6)	46 (29.5)	18 (20)	13 (26)	0.435
DM, n (%)	142 (38.2)	31 (40.8)	66 (42.3)	30 (33.3)	15 (30)	0.301
Smoking, n (%)	115 (30.9)	21 (27.6)	54 (34.6)	27 (30)	13 (26)	0.576
Clinical characteristics						
Creatinine (umol/L), Median (IQR)	73.0 (61.0, 90.0)	72.5 (61.8, 95.0)	74.5 (61.8, 91.2)	73.5 (63.0, 89.8)	70.0 (60.0, 84.2)	0.593
GPT (IU/L), Median (IQR)	23.0 (16.0, 36.2)	23.0 (16.8, 35.0)	23.0 (15.8, 40.2)	22.0 (15.2, 32.0)	25.0 (18.0, 40.0)	0.359
FPG (mmol/L), Median (IQR)	6.2 (5.0, 8.3)	6.6 (5.2, 8.5)	6.2 (5.0, 8.6)	6.1 (4.9, 7.8)	5.6 (4.8, 7.2)	0.193
HGB (g/L), Median (IQR)	124.0 (112.0, 133.2)	124.5 (117.5, 133.0)	124.5 (112.0, 135.2)	122.5 (112.2, 131.0)	126.0 (111.5, 136.0)	0.453
TC (mmol/L), Median (IQR)	3.7 (3.1, 4.4)	3.6 (3.1, 3.9)	3.8 (3.1, 4.4)	3.8 (3.3, 4.7)	3.7 (3.2, 4.6)	0.141
TG (mmol/L), Median (IQR)	1.2 (1.0, 1.7)	1.2 (0.9, 1.6)	1.3 (1.0, 1.7)	1.2 (1.0, 1.6)	1.1 (1.0, 1.6)	0.536
LDL-C (mmol/L), Median (IQR)	1.8 (1.4, 2.4)	1.7 (1.3, 2.1)	1.8 (1.5, 2.4)	1.9 (1.5, 2.7)	1.8 (1.4, 2.3)	0.060
LVEF (%), Median (IQR)	61.0 (50.0, 67.2)	62.0 (51.8, 68.0)	60.0 (48.0, 68.0)	59.5 (48.2, 67.0)	62.0 (56.2, 68.0)	0.178
LVEF ≤40%, n (%)	39 (10.5)	6 (7.9)	20 (12.8)	11 (12.2)	2 (4)	0.264
P2Y12 antagonists						0.541
Clopidogrel, n (%)	129 (34.7)	26 (34.2)	56 (35.9)	34 (37.8)	13 (26)	
Ticagrelor, n (%)	243 (65.3)	50 (65.8)	100 (64.1)	56 (62.2)	37 (74)	

Data are expressed as the mean ± SD or number (percentage). CVD indicates Cardio Vascular Diseases; SCAD, stable coronary heart disease; UA, unstable angina; STEMI, ST-segment elevation myocardial infarction; NSTEMI, non ST-segment elevation myocardial infarction; GPT, glutamic-pyruvic transaminase; FPG, fasting plasma glucose; HGB, hemoglobin; TC, total cholesterol; TG, triglyceride; LDL-C, low density lipoprotein C; LVEF, left ventricular ejection fraction.

**Table 2.** **Lesion, and procedural characteristics between different groups**.

Variables		Total (n = 372)	Group 1 (n = 76)	Group 2 (n = 156)	Group 3 (n = 90)	Group 4 (n = 50)	*p* value
Lesion characteristics							
Target vessel							
	LAD, n (%)	292 (78.5)	62 (81.6)	124 (79.5)	69 (76.7)	37 (74)	0.730
	LCX, n (%)	22 (5.9)	4 (5.3)	7 (4.5)	5 (5.6)	6 (12)	0.300
	RCA, n (%)	58 (15.6)	10 (13.2)	25 (16)	16 (17.8)	7 (14)	0.852
Ostial stenosis, n (%)		95 (25.5)	17 (22.4)	41 (26.3)	19 (21.1)	18 (36)	0.235
Proximal lesion, n (%)		346 (93.0)	71 (93.4)	144 (92.3)	85 (94.4)	46 (92)	0.920
Midcourse lesion, n (%)		321 (86.3)	66 (86.8)	138 (88.5)	73 (81.1)	44 (88)	0.423
Distal lesion, n (%)		143 (38.4)	34 (44.7)	53 (34)	31 (34.4)	25 (50)	0.111
Bifurcation, n (%)		26 (7.0)	1 (1.3)	11 (7.1)	8 (8.9)	6 (12)	0.071
Distortion, n (%)		24 (6.5)	2 (2.6)	10 (6.4)	8 (8.9)	4 (8)	0.352
Total lesion length (mm), Median (IQR)		71.5 (57.0, 91.2)	65.5 (54.8, 88.5)	80.0 (60.0, 98.0)	70.0 (56.0, 91.0)	67.0 (56.2, 90.0)	0.071
Procedural Characteristics							
IABP suport, n (%)		46 (12.4)	7 (9.2)	21 (13.5)	12 (13.3)	6 (12)	0.812
Number of burrs used							0.355
	1	356 (95.7)	72 (94.7)	147 (94.2)	87 (96.7)	50 (100)	
	2	16 (4.3)	4 (5.3)	9 (5.8)	3 (3.3)	0 (0)	
Initial burr size (mm), n (%)							0.125
	1.25	87 (23.4)	17 (22.4)	29 (18.6)	24 (26.7)	17 (34)	
	1.5	285 (76.6)	59 (77.6)	127 (81.4)	66 (73.3)	33 (66)	
Maximum burr size (mm), n (%)							0.096
	1.25	77 (20.7)	14 (18.4)	24 (15.4)	22 (24.4)	17 (34)	
	1.5	275 (73.9)	57 (75)	121 (77.6)	66 (73.3)	31 (62)	
	1.75	20 (5.4)	5 (6.6)	11 (7.1)	2 (2.2)	2 (4)	
Initial burr-to-artery ratio		0.5 ± 0.1	0.5 ± 0.1	0.5 ± 0.1	0.5 ± 0.0	0.5 ± 0.1	0.520
Maximum burr-to-artery ratio		0.5 ± 0.1	0.5 ± 0.1	0.5 ± 0.1	0.5 ± 0.0	0.5 ± 0.1	0.173
Maximum burr-to-artery ratio							0.590
	<0.6	360 (96.8)	73 (96.1)	152 (97.4)	88 (97.8)	47 (94)	
	≥0.6	12 (3.2)	3 (3.9)	4 (2.6)	2 (2.2)	3 (6)	
Initial rotational speed (× 1000 rpm), n (%)							<0.001
	13	17 (4.6)	17 (22.4)	0 (0)	0 (0)	0 (0)	
	14	59 (15.9)	59 (77.6)	0 (0)	0 (0)	0 (0)	
	15	168 (45.2)	0 (0)	156 (100)	0 (0)	12 (24)	
	16	117 (31.5)	0 (0)	0 (0)	90 (100)	27 (54)	
	18	11 (3.0)	0 (0)	0 (0)	0 (0)	11 (22)	
Higher speed than initial speed (× 1000 rpm), n (%)							<0.001
	0	323 (86.8)	76 (100)	156 (100)	90 (100)	1 (2)	
	1	7 (1.9)	0 (0)	0 (0)	0 (0)	7 (14)	
	2	38 (10.2)	0 (0)	0 (0)	0 (0)	38 (76)	
	3	1 (0.3)	0 (0)	0 (0)	0 (0)	1 (2)	
	4	3 (0.8)	0 (0)	0 (0)	0 (0)	3 (6)	
Maximum rotational speed (× 1000 rpm)		15.4 ± 1.4	13.8 ± 0.4	15.0 ± 0.0	16.0 ± 0.0	18.2 ± 1.2	<0.001
TIMI flow grade following RA, n (%)							0.006
	0	0 (0)	0 (0)	0 (0)	0 (0)	0 (0)	
	1	3 (0.8)	0 (0)	0 (0)	0 (0)	3 (6)	
	2	82 (22)	17 (22.4)	27 (17.3)	22 (24.4)	16 (32)	
	3	287 (77.2)	59 (77.6)	129 (82.7)	68 (75.6)	31 (62)	
Procedural success, n (%)		370 (99.5)	76 (100)	156 (100)	89 (98.9)	49 (98)	0.239
RA + drug-eluting stent, n (%)		368 (98.9)	76 (100)	156 (100)	89 (98.9)	47 (94)	0.008
RA + drug-coated ballon, n (%)		2 (0.5)	0 (0)	0 (0)	0 (0)	2 (4)	0.019
Emergent CABG, n (%)		2 (0.5)	0 (0)	0 (0)	1 (1)	1 (2)	0.237
Mean diameter of stents (mm), Median (IQR)		2.9 (2.8, 3.1)	3.0 (2.8, 3.2)	2.9 (2.8, 3.1)	2.9 (2.8, 3.1)	3.0 (2.8, 3.1)	0.622
Number of stents used, n (%)							0.172
	0	4 (1.1)	0 (0)	0 (0)	1 (1.1)	3 (6)	
	1	25 (6.7)	3 (3.9)	9 (5.8)	9 (10)	4 (8)	
	2	137 (36.8)	36 (47.4)	52 (33.3)	32 (35.6)	17 (34)	
	3	139 (37.4)	25 (32.9)	60 (38.5)	36 (40)	18 (36)	
	4	53 (14.2)	10 (13.2)	29 (18.6)	9 (10)	5 (10)	
	5	11 (3.0)	1 (1.3)	5 (3.2)	3 (3.3)	2 (4)	
	6	3 (0.8)	1 (1.3)	1 (0.6)	0 (0)	1 (2)	
Mean diameter of stents (mm), Median (IQR)		2.9 (2.8, 3.1)	3.0 (2.8, 3.2)	2.9 (2.8, 3.1)	2.9 (2.8, 3.1)	3.0 (2.8, 3.1)	0.622
Total stent length (mm), Median (IQR)		75.0 (59.0, 95.0)	69.0 (56.5, 91.5)	84.0 (62.0, 102.0)	74.0 (58.5, 95.0)	69.0 (58.2, 94.8)	0.090

Data are expressed as the mean ± SD or number (percentage). LAD, Left anterior descending artery; LCX, Left circumflex artery; RCA, Right coronary artery; IABP, Intra Aortic Balloon Pump; CABG, Coronary artery bypass grafting.

**Fig. 1.**
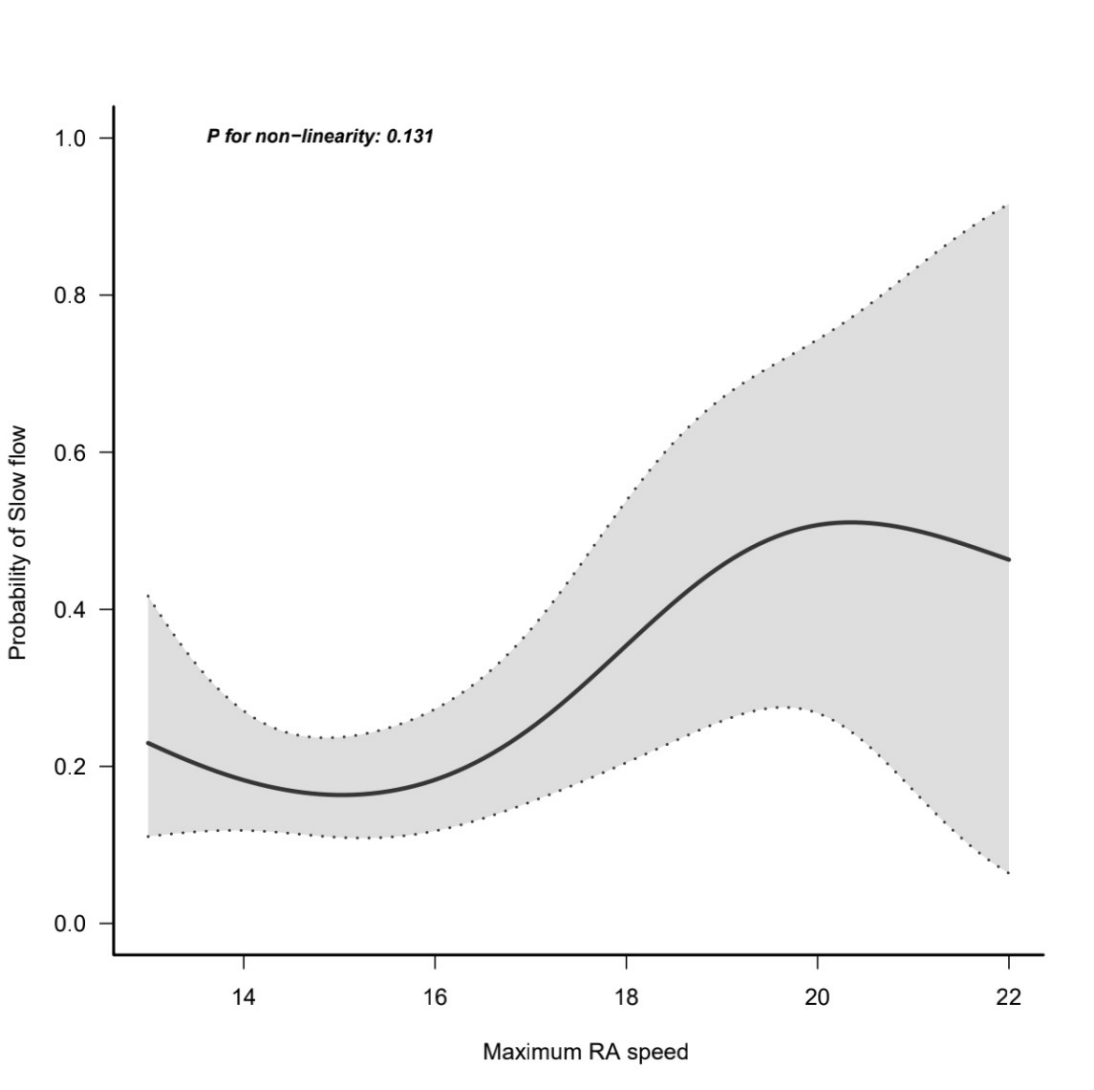
**The curvilinear relationship between the rotational speed and the probability of slow flow**.

In addition, we analyzed the incidence of complications during PCI as shown in Table 3. A total of 19 patients (38%) in group 4 experienced slow flow after RA, compared to 22.4%, 17.3% and 24.4% in group 1, group 2 and group 3, respectively (*p* = 0.025). A generalized additive model was used to assess the nonlinear relationship between the maximum rotational speed and incident of slow flow. A curvilinear relationship between the rotational speed and the probability of slow flow was developed as shown in Fig. 1. After adjusting for confounding factors such as age, sex or CVD history, the probability of slow flow was intensified with the increase in rotational speed (*p* for non-linearity = 0.131; adjusted model). A total of 48 patients (12.9%) developed vasospasm, among which 15 cases were in group 1, which was higher compared to the other three groups. However, there was no significant difference in the development of vasospasm among the four groups (*p* = 0.052). Moreover, the incidence of hypotension, dissection, perforation and bradyarrhythmia were comparable among the four groups (*p *> 0.05). There was occurrence of only 1 case of burr entrapment during RA in group 2, and the difference among the four groups was not statistically significant (*p* = 1.000). Furthermore, none of the four groups experienced rotawire fractures. There was also no significant difference in complications such as heart failure, stent thrombosis, and cardiac death, among the four groups (*p *> 0.05).

**Table 3.** **Comparison of in-hospital outcomes in different groups**.

Variables	Total	Group 1	Group 2	Group 3	Group 4	*p*
	(n = 372)	(n = 76)	(n = 156)	(n = 90)	(n = 50)	
Hypotension, n (%)	6 (1.6)	1 (1.3)	5 (3.2)	0 (0)	0 (0)	0.244
Vasospasm, n (%)	48 (12.9)	15 (19.7)	22 (14.1)	5 (5.6)	6 (12)	0.052
Dissection, n (%)	35 (9.4)	11 (14.5)	12 (7.7)	7 (7.8)	5 (10)	0.368
Slow flow, n (%)	85 (22.8)	17 (22.4)	27 (17.3)	22 (24.4)	19 (38)	0.025
Perforation, n (%)	7 (1.9)	2 (2.6)	3 (1.9)	1 (1.1)	1 (2)	0.905
Bradyarrhythmias, n (%)	11 (3.0)	2 (2.6)	4 (2.6)	2 (2.2)	3 (6)	0.566
Burr entrapment, n (%)	1 (0.3)	0 (0)	1 (0.6)	0 (0)	0 (0)	1.000
RotaWire fracture, n (%)	0 (0)	0 (0)	0 (0)	0 (0)	0 (0)	1.000
Heart failure, n (%)	139 (37.4)	24 (31.6)	59 (37.8)	35 (38.9)	21 (42)	0.648
Stent thrombosis, n (%)	3 (0.8)	0 (0)	1 (0.6)	2 (2.2)	0 (0)	0.563
Cardiac death, n (%)	8 (2.2)	0 (0)	4 (2.6)	3 (3.3)	1 (2)	0.459

Data are expressed as the number (percentage).

**Fig. 2.**
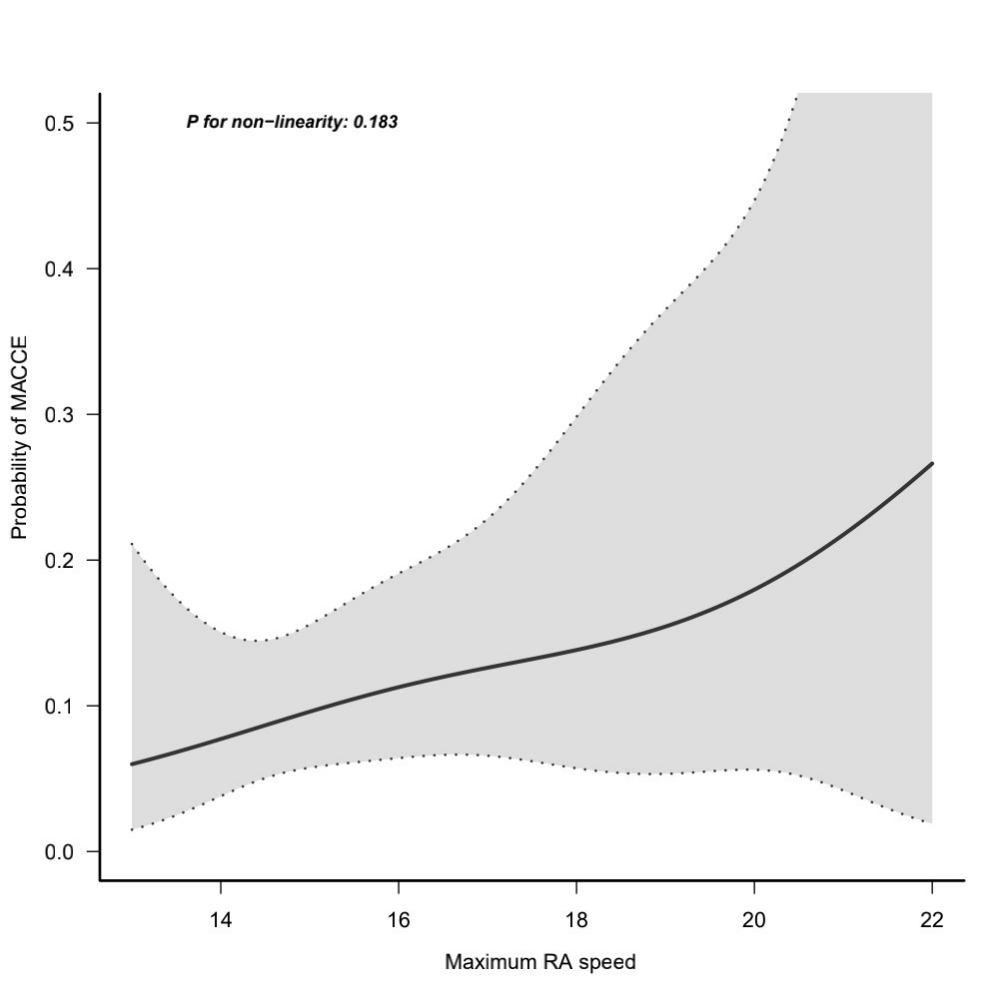
**The curvilinear relationship between the rotational speed and the probability of MACCE**. MACCE, major cardiovascular and cerebrovascular events.

**Fig. 3.**
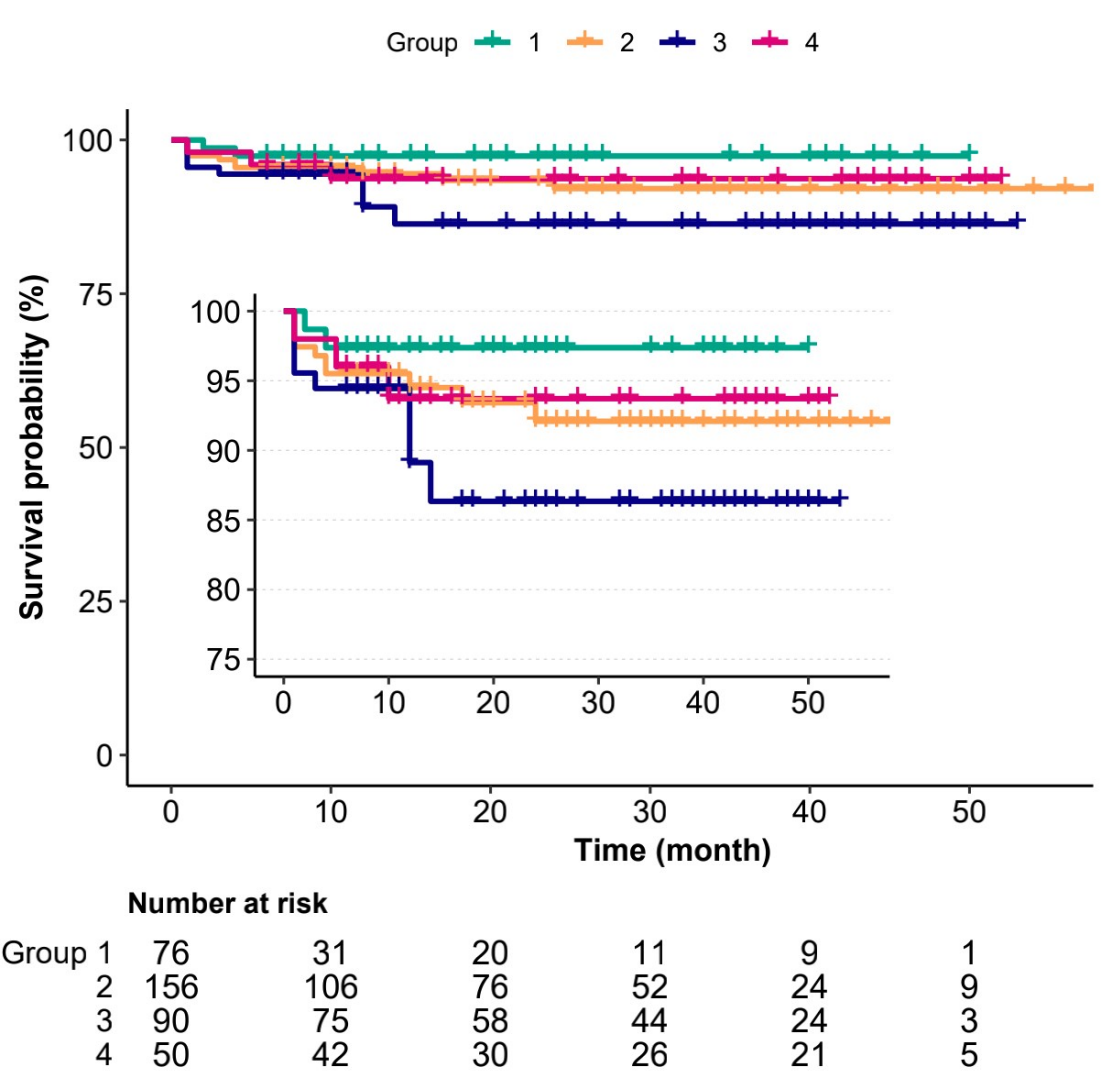
**Kaplan–Meier death from any reason-free survival curves**. Log rank *p* = 0.25. group 1 (<150,000 rpm), group 2 (150,000 rpm), group 3 (160,000 rpm), group 4 (≥170,000 rpm).

We analyzed the incidence of long-term endpoints in the four group as shown in Table 4. The data showed that there were 34 patients (9.1%) who experienced MACCE, among which 17 cases were in group 2, which was higher compared to the other three groups. However, no significant difference was observed among the four groups (*p* = 0.452). After adjustment for confounding factors, the probability of MACCE was intensified with the increase in rotational speed (*p* for non-linearity = 0.183; adjusted model) (Fig. 2). Analysis of mortality rate showed comparable six-months mortality among the four groups. However, there was no six-month follow-up cardiac deaths in group 1 (0%), and a lower rate of death from any reason (2.6%), which had no significant difference among the four groups (*p* = 0.288 and *p* = 0.832, respectively). Kaplan–Meier curves were plotted to assess survival data for patients with different rotational speed of the RA (Fig. 3). The log-rank test demonstrated that there was no significant differences in the survival rate between the four groups, with only borderline trend in favor of patients using lower rotational speed (*p* = 0.25). In addition, a total of 44 patients (11.8%) developed heart failure, with comparable incidence among the four groups (group 1 vs. group 2 vs. group 3 vs. group 4: 15.8% vs. 10.9% vs. 10% vs. 12%, *p* = 0.668).

**Table 4.** **Comparison of six-month endpoints in different groups**.

Variables	Total	Group 1	Group 2	Group 3	Group 4	*p*
	(n = 372)	(n = 76)	(n = 156)	(n = 90)	(n = 50)	
Primary endpoints						
MACCE, n (%)	34 (9.1)	4 (5.3)	17 (10.9)	7 (7.8)	6 (12)	0.452
MI, n (%)	3 (0.8)	0 (0)	1 (0.6)	0 (0)	2 (4)	0.100
Stent thrombosis, n (%)	8 (2.2)	0 (0)	7 (4.5)	1 (1.1)	0 (0)	0.097
TVR, n (%)	8 (2.2)	0 (0)	5 (3.2)	1 (1.1)	2 (4)	0.280
Cardiac death, n (%)	10 (2.7)	0 (0)	5 (3.2)	4 (4.4)	1 (2)	0.288
Death from any reason, n (%)	17 (4.6)	2 (2.6)	8 (5.1)	5 (5.6)	2 (4)	0.832
Stroke, n (%)	11 (3.0)	5 (6.6)	4 (2.6)	2 (2.2)	0 (0)	0.204
Secondary endpoints						
Heart failure, n (%)	44 (11.8)	12 (15.8)	17 (10.9)	9 (10)	6 (12)	0.668

Data are expressed as the number (percentage). MI, myocardial infarction; TVR, target vessel revascularization.

For the multivariable logistic regression analysis, we selected variables that commonly affect clinical cardiovascular outcomes such as age, sex or CVD history, as regression models (Table 5). After adjusting for age, sex, CVD history, FPG, glutamic-pyruvic transaminase (GPT), triglyceride (TG) and low density lipoprotein C (LDL-C), LVEF, maximum burr to artery ratio, as well as type of CHD, including SCAD, UA, ST-segment elevation myocardial infarction (STEMI), and NSTEMI in the analysis of vasospasm events, individuals with lower rotational speed (<150,000 rpm) were at 230% higher risk of vasospasm compared with a higher rotational speed (160,000 rpm) (OR = 3.3, 95% CI: 1.08~10.09, *p* = 0.036). Moreover, compared with a rotational speed of 150,000 rpm, individuals with higher rotational speed (≥170,000 rpm) were at 218% higher risk of slow flow (OR = 3.18, 95% CI: 1.52~6.63, *p* = 0.002). In addition, logistic regression analysis showed that rotational speed is a predictor of slow flow during RA operation (OR = 1.25, 95% CI: 1.05~1.49, *p* = 0.01), as well as a predictor of six-months incidence of stroke (OR = 0.5, 95% CI: 0.26~0.96, *p* = 0.036). There was no significantly increased risk for MACCE, TVR, cardiac death, death from any reason and heart failure events in individuals with different rotational speeds, compared to those with a rotational speed of 160,000 rpm.

**Table 5.** **Association between the speed of rotational atherectomy and clinical events: Multivariate Regression Analysis**.

	Variable	No. of total	No. of event (%)	Crude OR (95% CI)	*p* value	Adjusted OR (95% CI)	*p* value
Vasospasm	rotational speed	372	48 (12.9)	0.79 (0.62∼1.03)	0.077	0.85 (0.66∼1.1)	0.214
	<150,000 rpm	76	15 (19.7)	4.18 (1.44∼12.12)	0.008	3.3 (1.08∼10.09)	0.036
	150,000 rpm	156	22 (14.1)	2.79 (1.02∼7.65)	0.046	2.2 (0.77∼6.36)	0.138
	160,000 rpm	90	5 (5.6)	1 (Ref)		1 (Ref)	
	≥170,000 rpm	50	6 (12)	2.32 (0.67∼8.02)	0.184	2.11 (0.58∼7.62)	0.255
Slow flow	rotational speed	372	85 (22.8)	1.27 (1.07∼1.49)	0.005	1.25 (1.05∼1.49)	0.010
	<150,000 rpm	76	17 (22.4)	1.38 (0.7∼2.72)	0.357	1.61 (0.79∼3.26)	0.187
	150,000 rpm	156	27 (17.3)	1 (Ref)		1 (Ref)	1 (Ref)
	160,000 rpm	90	22 (24.4)	1.55 (0.82∼2.92)	0.179	1.56 (0.81∼3.01)	0.184
	≥170,000 rpm	50	19 (38)	2.93 (1.45∼5.93)	0.003	3.18 (1.52∼6.63)	0.002
MACCE	rotational speed	372	34 (9.1)	1.19 (0.95∼1.48)	0.131	1.14 (0.9∼1.44)	0.291
	<150,000 rpm	76	4 (5.3)	0.66 (0.19∼2.34)	0.519	0.9 (0.24∼3.44)	0.877
	150,000 rpm	156	17 (10.9)	1.45 (0.58∼3.64)	0.429	1.89 (0.71∼5.03)	0.201
	160,000 rpm	90	7 (7.8)	1 (Ref)		1 (Ref)	
	≥170,000 rpm	50	6 (12)	1.62 (0.51∼5.11)	0.413	1.9 (0.57∼6.32)	0.296
MI	rotational speed	372	3 (0.8)	1.84 (1.13∼2.99)	0.014	1.88 (0.82∼4.33)	0.139
	<150,000 rpm	76	0 (0)	1 (0∼Inf)	0.999	Inf (Inf∼Inf)	<0.001
	150,000 rpm	156	1 (0.6)	14986245.91 (0∼Inf)	0.996	Inf (Inf∼Inf)	<0.001
	160,000 rpm	90	0 (0)	1 (Ref)		1 (Ref)	
	≥170,000 rpm	50	2 (4)	96786171.49 (0∼Inf)	0.995	Inf (Inf∼Inf)	<0.001
Stent thrombosis	rotational speed	372	8 (2.2)	0.84 (0.47∼1.49)	0.549	0.8 (0.41∼1.58)	0.526
	<150,000 rpm	76	0 (0)	0 (0∼Inf)	0.994	0 (0∼0)	<0.001
	150,000 rpm	156	7 (4.5)	4.18 (0.51∼34.55)	0.184	4.89 (0.48∼50.21)	0.182
	160,000 rpm	90	1 (1.1)	1 (Ref)		1 (Ref)	
	≥170,000 rpm	50	0 (0)	0 (0∼Inf)	0.995	0 (0∼5.5632489495169e+66)	0.912
TVR	rotational speed	372	8 (2.2)	1.32 (0.9∼1.94)	0.152	1.5 (0.92∼2.47)	0.107
	<150,000 rpm	76	0 (0)	0 (0∼Inf)	0.994	0 (0∼1.03193924492696e+155)	0.953
	150,000 rpm	156	5 (3.2)	2.95 (0.34∼25.63)	0.327	3.92 (0.28∼55.42)	0.312
	160,000 rpm	90	1 (1.1)	1 (Ref)		1 (Ref)	
	≥170,000 rpm	50	2 (4)	3.71 (0.33∼41.96)	0.29	4.6 (0.25∼84.9)	0.305
Cardiac death	rotational speed	372	10 (2.7)	1.09 (0.72∼1.65)	0.674	1.13 (0.68∼1.89)	0.628
	<150,000 rpm	76	0 (0)	0 (0∼Inf)	0.989	0 (0∼Inf)	0.981
	150,000 rpm	156	5 (3.2)	0.71 (0.19∼2.72)	0.62	0.68 (0.13∼3.41)	0.635
	160,000 rpm	90	4 (4.4)	1 (Ref)		1 (Ref)	
	≥170,000 rpm	50	1 (2)	0.44 (0.05∼4.04)	0.467	0.48 (0.04∼6.36)	0.577
Death from any reason	rotational speed	372	17 (4.6)	1.09 (0.79∼1.5)	0.605	1.13 (0.79∼1.63)	0.497
	<150,000 rpm	76	2 (2.6)	0.46 (0.09∼2.44)	0.361	0.48 (0.08∼2.97)	0.43
	150,000 rpm	156	8 (5.1)	0.92 (0.29∼2.9)	0.885	0.96 (0.27∼3.39)	0.951
	160,000 rpm	90	5 (5.6)	1 (Ref)		1 (Ref)	
	≥170,000 rpm	50	2 (4)	0.71 (0.13∼3.79)	0.687	0.95 (0.16∼5.66)	0.958
Stroke	rotational speed	372	11 (3)	0.55 (0.3∼1)	0.049	0.5 (0.26∼0.96)	0.036
	<150,000 rpm	76	5 (6.6)	3.1 (0.58∼16.45)	0.184	3.53 (0.92∼13.47)	0.065
	150,000 rpm	156	4 (2.6)	1.16 (0.21∼6.45)	0.867	1.49 (0.43∼5.2)	0.529
	160,000 rpm	90	2 (2.2)	1 (Ref)		1 (Ref)	
	≥170,000 rpm	50	0 (0)	0 (0∼Inf)	0.992	0 (0∼2219057.84)	0.568
Heart failure	rotational speed	372	44 (11.8)	0.91 (0.72∼1.16)	0.467	0.9 (0.71∼1.14)	0.369
	<150,000 rpm	76	12 (15.8)	1.69 (0.67∼4.25)	0.267	1.73 (0.64∼4.63)	0.277
	150,000 rpm	156	17 (10.9)	1.1 (0.47∼2.58)	0.826	1.04 (0.43∼2.55)	0.931
	160,000 rpm	90	9 (10)	1 (Ref)		1 (Ref)	
	≥170,000 rpm	50	6 (12)	1.23 (0.41∼3.67)	0.714	1.22 (0.39∼3.87)	0.731

Multifactor models adjusted for SCAD, UA, NSTEMI, STEMI, sex, age, CVD history, FPG, GPT, TG, LDL-C, LVEF and maximum burr to artery ratio. SCAD, stable coronary heart disease; UA, unstable angina; STEMI, ST-segment elevation myocardial infarction; NSTEMI, non ST-segment elevation myocardial infarction; CVD Cardio Vascular Diseases; FPG, fasting plasma glucose; TG, triglyceride; LDL-C, low density lipoprotein C; GPT, glutamic-pyruvic transaminase; MI, myocardial infarction; TVR, target vessel revascularization.

## 4. Discussion

In our study, we investigated interventional outcomes of RA at different rotational speeds and compared clinical outcomes associated with RA in CHD patients. Our findings demonstrated that CHD patients treated with high rotational speeds (≥170,000 rpm) had a higher risk of slow flow events after RA. There was no significant difference in six-month outcomes in comparison to elective CHD patients with different rotational speeds, while the probability of MACCE was intensified with increase in rotational speed. A rotational speed of <150,000 rpm was shown to be an independent risk factor for spasm during RA in the CHD patients. Lastly, our data demonstrated that rotational speed is an independent risk factor for slow flow and six-month MI in CHD patients.

RA speed remains an important issue because of its standardization challenges among different RA surgeons. Expert consensus, including in China, Japan and Europe, has differential recommendations for RA speed [9, 10, 11, 12, 13]. For instance, the European RA expert consensus recommends a safe range of rotablation speed between 135,000 and 180,000 rpm [9]. On the other hand, the consensus of Chinese RA experts recommended a starting speed of 135,000~180,000 rpm. However, for lesions that cannot completely pass through the stenosis after repeated operations, the speed can be increased to a maximum of 220,000 rpm [13]. However, the expert consensus on RA does not refer to whether speed of rotablation is associated with the long-term prognosis. Moreover, data on long-term effect of RA remain scant. Here, we performed a targeted study to evaluate the outcomes of RA speed in CHD patients.

The rotational speed used during RA was between 130,000–220,000 rpm for all the patients in this study, which is in line with recommendation of most of the current guidelines. Our analysis showed that the overall incidence of slow flow was 21%, which was relatively higher than that observed in the randomized ROTAXUS and PREPARE-CALC trials [14, 15]. This may be due to the low proportion of ACS in ROTAXUS and PREPARE-CALC trials, and their target lesion length was significantly shorter (27.7 ± 12.2 mm and 29.81 ± 15.23, respectively), compared to that in our study. By grouping the differences in rotational speed and comparing the differences in perioperative complications, it was shown that patients in the high rotational speed group (≥170,000 rpm) experienced significantly higher incidence of slow flow. Compared with a rotational speed of 150,000 rpm, individuals with a rotational speed of ≥170,000 rpm were at a 218% higher risk of slow flow. This may be associated with the fact that high rotational speed of RA can easily induce thermal damage and platelet activation during RA [16, 17, 18]. Thermal injury is associated with platelet activation, smooth muscle proliferation and coronary restenosis. Another study showed that reduction of the rotational speed of RA suppress platelet activation [19]. In contrast, Sakakura *et al*. [6] did not show any benefit associated with low rotational speed (140,000 rpm) in preventing slow flow in RA. In this prospective, randomized, single-center study, patients were randomized to low-speed (140,000 rpm) or high-speed (190,000 rpm) RA. It is worth noting that in clinical practice, operators cannot predict a fixed speed that would pass through calcified lesions, thus this grouping scheme has limitations in clinical practice. The RA protocol used in our study was more reasonable and our study included patients with STEMI and NSTEMI, which were excluded from the analysis in the previous study [6]. In addition, our study had a larger sample size compared to Sakakura *et al*. [6], which had only 100 cases. Moreover, the burr size used in our study was larger. A smaller burr size minimizes platelet aggregation caused by RA and may also reduce distal embolisation simply by less luminal gain, which might reduce the incidence of slow flow.

Like the risk of slow flow during PCI, highest six-month MACCE occurrence in CHD patients with rotational speed (≥170,000 rpm) and the probability of MACCE was intensified with the increase in rotational speed. This might also be related to the fact that the rotational speed of RA easily induces platelet activation, thermal damage, and slow flow. Platelet activation is an important factor that would affect the outcome of patients undergoing PCI. Our study showed that there was no significant differences in the occurence of six-month TVR in CHD patients with different rotational speed. Uetani *et al*. [20] demonstrated that RA with a low platform speed (150,000–160,000 rpm) can be performed and yield a lower incidence of 1-year restenosis compared to a high platform speed (170,000–190,000 rpm). Unfortunately, endovascular imaging such as IVUS and optical coherence tomography (OCT), was not performed on all patients in our study, and thus data such as minimum lumen area was lacking. In addition, logistic regression analysis showed that rotational speed is a predictor of six-months incidence of stroke. These results may be related to the individual differences of the selected patients, which include own cerebrovascular conditions. Thus, there is a need for further studies.

The incidence of vasospasm in the group with a rotational speed of <150,000 rpm was higher than in the other three groups. Compared with a rotational speed of 160,000 rpm, individuals with lower rotational speed (<150,000 rpm) were at 230% higher risk of vasospasm. We speculate that this may be associated with the longer contact time between the burr and the calcified plaque per unit time in the lower speed group, which is more likely to induce vasospasm. In our experience with RA, vasospasm after RA may lead to a transient drop in blood pressure and heart rate, but a large proportion of the patients only experienced a mild drop in blood pressure and heart rate, which did not meet the definition criteria in our study. In addition, once vasospasm is found by the surgeon after RA, nitroglycerin, verapamil or the other dilators will be injected into the coronary artery as soon as possible to avoid the continuous occurrence of vasospasm. This may be the reason why the rates of hypotension and bradyarrhythmia in this study are so low.

Although our study highlights important findings, it still has some limitations that need to be considered. Firstly, it is a single-center, retrospective analysis, which cannot represent a randomized controlled trial. Secondly, the sample size was relatively small, with a short follow-up time which is underpowered for all clinical outcomes especially for the long term outcomes. In addition, there was also lack of total duration time of RA, lack of routine use of intravascular ultrasound or optical coherence tomography to optimize interventional procedures. Prospective randomized controlled trials with larger sample sizes are needed to further confirm the findings.

## 5. Conclusions

In conclusion, CHD patients treated with RA at a rotational speed of ≥170,000 rpm had a higher risk of slow flow after RA. Rotational speed was an independent risk factor for slow flow in CHD patients. A rotational speed of <150,000 rpm was associated with a higher risk of vasospasm compared with rotational speed of 160,000 rpm. There was no significant difference in six-month outcomes in comparison to elective CHD patients with different rotational speeds, while the probability of MACCE was intensified with the increase in rotational speed.

## Data Availability

All data used or analysed during the current study are available from the corresponding author on reasonable request.
